# Can Mild-to-Moderate Iodine Deficiency during Pregnancy Alter Thyroid Function? Lessons from a Mother–Newborn Cohort

**DOI:** 10.3390/nu14245336

**Published:** 2022-12-15

**Authors:** Simon Shenhav, Carlos Benbassat, Dov Gefel, Shmuel Zangen, Shani R. Rosen, Yael Avrahami-Benyounes, Shlomo Almashanu, Ludmila Groisman, Efrat Rorman, Shlomo Fytlovich, Eyal Y. Anteby, Yaniv S. Ovadia

**Affiliations:** 1 Obstetrics and Gynecology Department, Barzilai University Medical Center, Ashkelon 7830604, Israel; 2 Faculty of Health Sciences, Ben-Gurion University of Negev, Beersheba 8410501, Israel; 3 Endocrine Institute, Shamir Medical Center (Assaf Harofeh), Be’er Ya’akov 7033001, Israel; 4 Sackler Faculty of Medicine, Tel Aviv University, Tel Aviv 6997801, Israel; 5 School of Nutritional Science, Institute of Biochemistry, Food Science and Nutrition, Robert H. Smith Faculty of Agriculture, Food and Environment, The Hebrew University of Jerusalem, Rehovot 76100001, Israel; 6 Department of Neonatology, Barzilai University Medical Center, Ashkelon 7830604, Israel; 7 Women’s Health Center, Maccabi Healthcare Services, Southern Region, Beersheba 8489312, Israel; 8 National Newborn Screening Program, Ministry of Health, Tel-Hashomer 5262000, Israel; 9 National Public Health Laboratory, Ministry of Health, Tel Aviv 6108401, Israel; 10 Laboratory of Clinical Biochemistry, Barzilai University Medical Center, Ashkelon 7830604, Israel; 11 Foreign Studies Department, Robert H. Smith Faculty of Agriculture, Food and Environment, The Hebrew University of Jerusalem, Rehovot 76100001, Israel

**Keywords:** free triiodothyronine, iodine insufficiency, maternal dietary iodine intake, neonatal thyrotropin, thyroglobulin

## Abstract

Severe iodine deficiency during pregnancy has substantial hormonal consequences, such as fetal brain damage. Data on the potential effects of mild-to-moderate iodine deficiency on the thyroid function of pregnant women and their newborns are scarce and divergent. We investigated the association between iodine intake in pregnancy and maternal and neonatal thyroid function in a region with mild-to-moderate iodine deficiency. Pregnant women’s iodine status was evaluated using an iodine food frequency questionnaire, serum thyroglobulin (Tg), and urinary iodine concentration (UIC). Neonatal thyrotropin (nTSH) values were measured after birth. Obstetrics and anthropometric data were also collected. Among the 178 women (median age 31 years) included in the study, median (interquartile range) estimated dietary iodine intake, Tg and UIC were 179 (94–268) μg/day, 18 (11–33) μg/L, and 60 (41–95) μg/L, respectively. There was a significant inverse association of iodine intake with Tg values among the study population (β = −0.2, F = 7.5, *p* < 0.01). Women with high free triiodothyronine (FT3) values were more likely to exhibit an estimated iodine intake below the estimated average requirement (160 μg/day, odds ratio [OR] = 2.6; 95% confidence interval [CI], 1.1–6.4; *p* = 0.04) and less likely to consume iodine-containing supplements (OR = 0.3, 95% CI, 0.1–0.8; *p* = 0.01). It is possible that thyroid function may be affected by iodine insufficiency during pregnancy in regions with mild-to-moderate iodine deficiency. The relatively small sample size of the studied population warrants further investigation.

## 1. Introduction

Iodine is essential for the synthesis of the thyroid hormones triiodothyronine (T3) and thyroxine (T4). During pregnancy, the iodine demand rises to compensate for increased renal clearance and to support normal fetal growth and development. According to the World Health Organization (WHO), iodine deficiency in pregnant populations occurs if the median urinary iodine concentration (UIC) is <150 μg/L [1]. According to the American Thyroid Association (ATA) guidelines, mild-to-moderate iodine deficiency is defined if the median UIC is 50–150 µg/L [2]. The 150 µg/L threshold is based on theoretical assumptions regarding absorption, metabolic needs, and urinary excretion among pregnant women rather than on observed birth or pregnancy outcomes (including maternal and neonatal thyroid function), limiting its use in pregnancy assessment.

Mild-to-moderate iodine deficiency is prevalent in pregnant women living in developed countries. Recent national surveys have found median UICs of 144, 104, 66, 114, 118, and 61 µg/L in representative samples of pregnant women from the US, Portugal, North Ireland, Sweden, Greece, and Israel, respectively [3,4,5]. The consequences of severe iodine deficiency during pregnancy (defined as UIC < 50 μg/L) are well established and include maternal hypothyroidism, goiter, abortion, stillbirth, congenital hypothyroidism as well as adverse neurological and growth impairment in the offspring, including cretinism [1,6]. However, in mild-to-moderate maternal iodine deficiency, less is known about the potential outcomes. Moreover, the few data addressing this important concern are divergent—on the one hand, some randomized-controlled trials failed to identify the benefits of correcting maternal iodine deficiency in areas of mild-to-moderate deficiency; on the other hand, observational studies have shown that mild to moderate maternal iodine deficiency might negatively affect offspring neurodevelopment [7,8,9]. Therefore, solid evidence and proof regarding the association between mild-to-moderate maternal iodine deficiency and newborn health is lacking.

Clinical and biochemical data on maternal and neonatal thyroid function in regions with mild-to-moderate iodine deficiency are limited. The Ashkelon sub-district of Israel is a geographical region with an identified reliance on iodine-diluted desalinated seawater in a country without universal salt iodization (USI) [4,10,11,12]. In a recent national iodine survey, the iodine status among pregnant women residing in this sub-district was similar to that of the overall pregnant women population (64 μg/L vs. 61 μg/L, respectively), as assessed by median UIC [4]. Although WHO recommends USI as a method for achieving and maintaining adequate iodine intake in the public, Israel remains one of the last developed countries to have no policy mandating salt fortification [6,12]. It is remarkable that this policy gap exists even though iodine deficiencies (whether mild, moderate, or severe) affect populations at all levels of economic development [13]. Moreover, a recent systematic review and meta-analysis suggested that USI alone may not be enough to ensure iodine sufficiency during pregnancy [14]. In addition, the Israeli national biomonitoring program reported persistent population and maternal iodine deficiency (with a median UIC of 62.2 μg/L among 120 childbearing age women) and a low prevalence of self-reported iodized salt use among adults (5.4%, *N* = 233) [12]. Given the possible maternal and neonatal thyroid dysfunction due to mild-to-moderate iodine deficiency, we aimed to investigate and describe the association between iodine intake in pregnancy and both maternal and neonatal thyroid function in this population.

## 2. Materials and Methods

### 2.1. Participants, Settings, and Design

**Figure 1:**
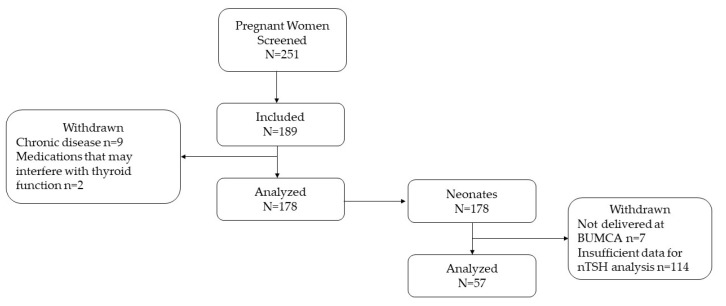
Study flow chart. Abbreviations: BUMCA = Barzilai University Medical Center, Ashkelon, nTSH = neonatal thyrotropin.

This study was conducted between June 2018 and April 2020. Pregnant women (*N* = 251) who attended the Obstetrics and Gynecology Department of Barzilai University Medical Center, Ashkelon, Israel (BUMCA), were enrolled and screened for study participation. Women were included in the study if they planned to deliver at BUMCA, were 20 years of age or older, were carrying a single fetus, and residing in the Ashkelon sub-district. Of the 189 women recruited to the study, 11 women were excluded due to documented chronic diseases (*N* = 9) and the use of medications that may interfere with thyroid function (*N* = 2). The detailed screening process, study sample generation, and follow-up flow chart are described in Figure 1.

The study was approved by BUMCA’s ethics committee (approval number 001-17-BRZ dated 7 August 2017). All participating pregnant women provided written informed consent after the research protocol was explained to them in detail. All dietary interviews were performed by experienced registered dietitians (RDs) authorized by the Israeli Ministry of Health. The assessing research group was blinded to individual participant iodine intake. Medical file screening, interviewing, and individual participant iodine intake was conducted using a coding system to ensure validity.

### 2.2. Data Collection

Obstetrics data (parity, gravidity, in vitro fertilization treatments, gestational week at recruitment, and anthropometrics) were collected from the participants’ medical files. The participants were interviewed using a structured sociodemographic, health, and habits questionnaire that was based on a previously validated questionnaire from the Israeli National Nutrition and Health Survey [15]. The interviews were conducted by three of the authors (YSO, SRR, and YAB), all of whom are RDs with experience interviewing pregnant women using validated questionnaires. The questionnaire included a series of multiple-choice and open-ended questions to collect information on demographic characteristics that could influence dietary and lifestyle practices. Self-reported health status and knowledge, attitudes, and behaviors regarding nutrition and health were also included in the questionnaire. Smoking prior to conception and during pregnancy was ascertained through interview-administered questionnaires. Pre-pregnancy body mass index (BMI) was calculated based on weight and height obtained from electronic medical records (82% of cases when data regarding routine visits between pregnancies was available) or self-report after recruitment (18% of cases when data regarding routine visits between pregnancies was not available).

### 2.3. Assessment of Maternal Iodine Levels

In order to assess the participants’ iodine status, four types of iodine measurements were performed: (a) long-term (up to one year) iodine intake using a validated, semi-quantitative iodine food frequency questionnaire (sIFFQ); (b) serum thyroglobulin (Tg) indicating intermediate-term iodine intake (weeks to months) [11]; (c) UIC as an indicator of iodine intake in recent days; and (d) neonate thyrotropin (nTSH) values to determine iodine sufficiency [6].

The sIFFQ was designed to estimate participants’ daily iodine intake in the year prior to the administration of the questionnaire. This sIFFQ outline was adapted and translated from a similar validated questionnaire [16] with local modifications and review by three of the authors (YSO, SRR, and YAB). The sIFFQ was pilot tested in a representative sample of pregnant women for readability, clarity of instruction, ease of administration, and time needed for completion. It contained questions regarding the average frequency and amounts of 28 selected foods with relatively high iodine content, which Israelis generally consume. The interview was conducted only by RDs (YSO, SRR, and YAB), and the participants were not informed that the purpose of the interview was to estimate iodine intake. Food models, measuring tools, and photographs were provided when necessary to reduce variation among interviewees. To deal with recall bias regarding supplement daily intake and iodized salt, the sIFFQ included questions on both initiation and time-frame consumption of iodine-containing supplements as well as the number of estimated teaspoons or the number of salt shaker shakings per consumed food. Follow-up phone calls to interviewees were made to clarify information (e.g., for checking the manufacturer’s label for iodine amounts in a dietary supplement). In order to avoid calculation errors and to improve estimation, a formula was implemented into a computerized algorithm using Excel software, version 16 (Microsoft Cooperation, Santa Rosa, CA, USA), to weigh the dietary information that was drawn for each food item included in the sIFFQ. The mean estimated daily dietary intake level was calculated as follows:

I = ∑ Fi × Qi × Ci

I represents iodine intake, ∑ is the amount or number of food items, F is the frequency per day, i signifies the food items, Q is the quantity or serving size, and C is the iodine content in the food.

The estimates of the food iodine content, which probably covers more than 90% of the participants’ total iodine amount consumed per day [17,18], were derived from multiple sources: iodine-containing supplements and iodized salt from the manufacturer labels; freshwater fish from the Agricultural Service of Israel and the Israeli Fish Breeders Association [19]; milk products (including milk, cheese, and yogurt) [18]; specific estimates of iodine concentration in tap water according to geographic locality [10]; foods of marine origin (including fish and seafood); and other selected sources as described elsewhere [10,20]. The participants were classified as having adequate estimated iodine intake when their sIFFQ calculated result was ≥160 μg/day, which is the estimated average requirement (EAR) for iodine intake by pregnant women [21]. The participants were also interviewed about selected daily dietary goitrogen exposure (pine, almonds, millet, linseeds, sorghum, sweet potato, cabbage, kale, cauliflower, and broccoli).

To determine thyroid function, the participants provided non-fasting venous blood samples within 24 h of recruitment. The samples were centrifuged immediately, and the serum was separated and stored at −20 °C until analysis. Free thyroxine (FT4), free triiodothyronine (FT3), thyrotropin (TSH), thyroid peroxidase antibodies (TPOAb), thyroglobulin antibody (TgAb), and Tg were analyzed by the clinical biochemistry laboratory at BUMCA using electrochemiluminescence immunoassay on the Modular Analytics E601 analyzer (Cobas, Roche Diagnostics GmbH, Mannheim, Germany). Reference ranges were 0.27–4.2 mU/L for TSH and 0.93–1.7 ng/dL for FT4, according to the manufacturer. TgAb above 40 IU/mL and TPOAb above 35 IU/mL were considered positive, as reported in previous studies [22]. In the absence of a consensus regarding the definition of isolated hypothyroxinemia (IHT) and established trimester-specific thyroid function tests values from iodine-deficient areas, reference intervals of FT4 from BUMCA in conjunction with trimester-specific cutoffs for TSH (2.5 mU/L for the first trimester, and 4.0 mU/L for both the second and third trimesters) were used to define IHT at different gestational ages [2]. Accordingly, the participants were classified as having subclinical hypothyroidism when their TSH values were above the TSH trimester-specific cutoffs. Despite the lack of consensus on normal Tg values during pregnancy [8], we considered iodine status as sufficient when Tg concentrations were <20 µg/L as previously demonstrated among a representative sample of pregnant women of Mediterranean European origin with mild iodine deficiency [23]. In addition, Tg values >40 µg/L were considered abnormally high based on previous studies [8]. A median cutoff ≤13 μg/L for Tg levels was considered sufficient for the entire study sample according to a recently suggested standard for populations [20]. The normal reference range for FT3 was within the 2.5 to 97.5 percentiles of its distribution after excluding TPOAb/TgAb-positive, abnormal Tg values, and smokers.

A spot urine sample was collected into an iodine-free cup within 4 h of recruitment to assess UIC (μg/L). Each sample was centrifuged, and the urine specimen was drawn into vacutainer tubes containing stabilizers (chlorhexidine, ethylparaben, and sodium propionate) without dipstick testing that could otherwise contaminate the iodine assay. Then specimens were refrigerated and delivered to the National Biomonitoring Laboratory of the Israeli Ministry of Health for UIC analysis. UIC was determined by inductively coupled plasma mass spectrometry method (ICP-MS) according to the US CDC ICP-MS method [24] using an Agilent 7800 ICP-MS Instrument (Santa Clara, CA, USA), equipped with Integrated Sample Introducing System and High Matrix Introducing mode. The method’s limit of quantification (LOQ) of 2.5 μg/L was determined during the method validation step by independent measurements of spiked pooled samples. For this purpose, iodine-free urine was collected based on previous ICP analysis with no iodine signal detection above this in a purified water blank. Accuracy (as relative recovery) and precision (as RSD) for LOQ were within 70–130% and 30%, respectively. Accuracy and precision—calculated from the result obtained from purchased control material in the mid-range (20–150 µg/L) tested with each set of samples (one control material to each of ten samples)—were within 96–103% and 8%, respectively. Proficiency in the analytical procedure was established by successful participation in the EQUIP external quality control program of the United States Centers for Disease Control [25] and the German External Quality Assessment Scheme, G-EQUAS [26].

### 2.4. Neonatal Birth Data and Iodine Measurements

Information on the participants’ newborns’ date of birth, gender, Apgar score, birth weight, length, and head circumference was obtained from participants’ medical records at BUMCA. Gestational age at birth was calculated according to participants’ self-reported last menstrual period and was confirmed using fetal crown-rump length. This parameter was calculated in single numbers of days added to completed weeks. Newborn birth weight percentiles and length and head circumference percentiles were calculated and standardized according to the Israeli birth population index using gestational age and gender as described in detail elsewhere [27,28].

Blood from neonates was collected by a heel prick and blotted using a 903-filter paper-dried blood spot (DBS; Eastern Business Forms, Greenville, SC, USA). Routinely, DBS samples are analyzed in a centralized laboratory within 3–5 days after birth. For this study, only DBS samples that were collected more than 48 h after birth were included. Total T4 (TT4) and nTSH were measured by the Israeli National Newborn Screening Program using a sensitive commercial AutoDELFIA time-resolved fluoroimmunoassay (PerkinElmer, Wallac Oy, Finland). Neonatal TSH values >5 IU/L were considered elevated, and TSH > 20 IU were considered abnormal. Neonate thyrotropin (nTSH) values >5 lU/L in more than 3% of total collected samples taken 3–4 days after birth were used to define iodine sufficiency [6,11].

### 2.5. Statistical Analysis

Statistical analyses were performed with the JMP Pro software, version 15 (SAS Institute, Cary, NC, USA). Categorical parameters were summarized by number and percentage and were compared by group (classified by iodine intake or FT3 decile) using the chi-squared test, likelihood test, Pearson test, or Fisher’s exact test as appropriate. Odds ratios (OR) were also calculated for the likelihood of iodine intake < EAR or for consuming iodine-containing supplements across FT3 decile-selected groups. Continuous parameters that were not distributed normally were summarized by the median and interquartile range (IQR). Normally distributed continuous parameters were presented as mean ± standard deviation (SD). In order to test whether continuous parameters (such as estimated iodine intake levels, anthropometric measurement results, and thyroid function serum values) had a normal distribution, a Goodness-of-fit test was performed and determined by the Shapiro–Wilk W or Cramer–von Mises W tests. These parameters were compared by one-way analysis of variance (ANOVA) with Student’s *t* (for means), Welch’s ANOVA (for unequal variances), Kruskal–Wallis (for non-parametric parameters), and median test (for medians) when appropriate. Serum Tg values were log-transformed before the analysis in order to normalize the distribution. Geometric means of serum Tg values were calculated on back-transformed data. The association of continuous UIC levels and estimated iodine intakes by the sIFFQ, FT3, and log-transformed Tg values were determined by linear regression (adjusting for possible confounding parameters by multivariate analysis, when necessary).

The study’s sample size was determined according to the difference in neonatal thyroid function tests, including TT4, between newborns to mothers with inadequate iodine intake and newborns to mothers with adequate iodine intake. According to the literature and a recent report on an Israeli cohort [29,30,31,32,33], it was estimated that, at 2–5 days postpartum, the TT4 total mean would be 15 nmol/L with an SD of 4 μg/L. To detect a change of 2 nmol/L in TT4 between newborns of mothers with inadequate iodine intake and mothers with adequate iodine intake between recruitment and 2–5 days after delivery, with 80% power and 2-sided alpha of 0.05, it was estimated that 128 mother–child pairs would be needed for the study (considering two groups with possibly different distributions and different numbers of individuals). After accounting for approximately 33% attrition/dropout (due to loss to follow-up or delivery in another medical center), 178 mother–child pairs were recruited to the study.

## 3. Results

### 3.1. Study Population

Of 189 consecutive participants screened, 178 (median age, 31; range, 20–46 years) were eligible to participate in the study. Most participants (80.3%) were in the third trimester of pregnancy; the median gestational age (week + days) at recruitment was 32 + 1 (range, 4–41 weeks). The sociodemographic, obstetric, health, and iodine intake characteristics of the study group are shown in Table 1.

### 3.2. Maternal Characteristics by Iodine Status, Thyroid Function, and Pregnancy Outcomes

The sIFFQ was completed by all participants, and no nutritional results were excluded as a result of missing, incomplete, or implausible information. Dietary iodine intake as estimated by the sIFFQ was considered adequate in 74 participants (41.6%), with a median intake of 290 µg/day (IQR 253–338 µg/day), but was inadequate in 106 (58.45%) with a median intake of 111 µg/day (IQR 65–168 µg/day). The overall median estimated iodine intake was above the EAR (179 vs. 160 µg/day), and 108 (61%) reported intake of iodine-containing supplements. In addition, median Tg and UIC levels were below the cutoffs considered by the WHO for iodine sufficiency levels in pregnancy (Table 1).

**Figure 2:**
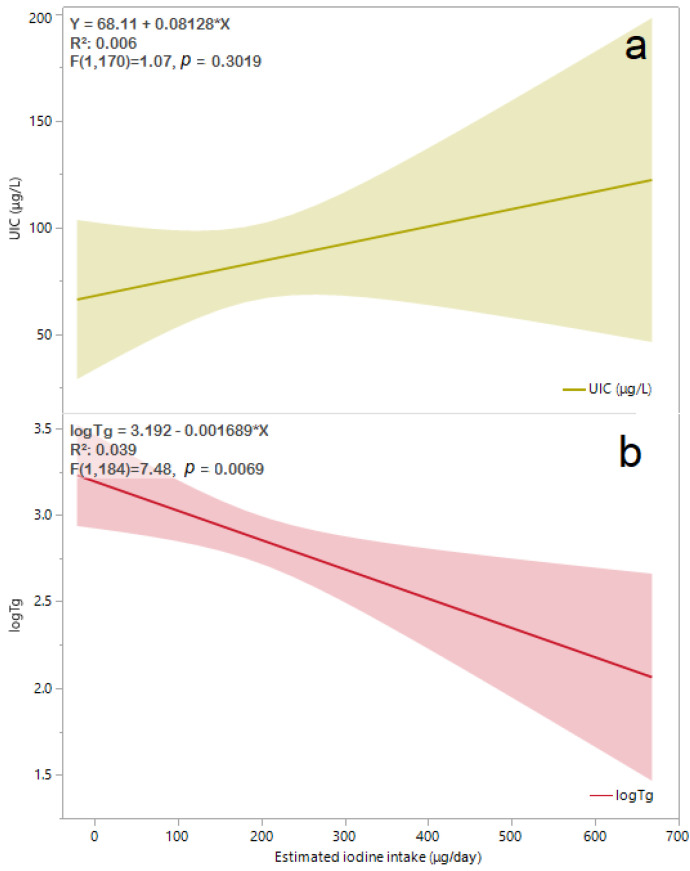
Available UIC (**a**) levels (*N* = 165) and logTg (**b**) values (*N* = 189) by estimated iodine intake obtained from sIFFQ. The regression line equation, R^2^ and *p* value for associations of UIC and logTg are shown in the left-upper area of (**a**) and (**b**), respectively. The solid line represents the estimated linear fit, and the shaded areas are the 95% CI. After adjusting for participants’ age, smoking status, and gravidity, the correlation between logTg and estimated iodine intake remained significant [β (95% CI) = −0.198 (−0.0005, −0.003), *p* = 0.04]. Abbreviations: UIC, urinary iodine concentration; sIFFQ, semi-quantitative iodine food frequency questionnaire; log Tg, serum log-transformed thyroglobulin; CI, confidence interval.

**Figure 3:**
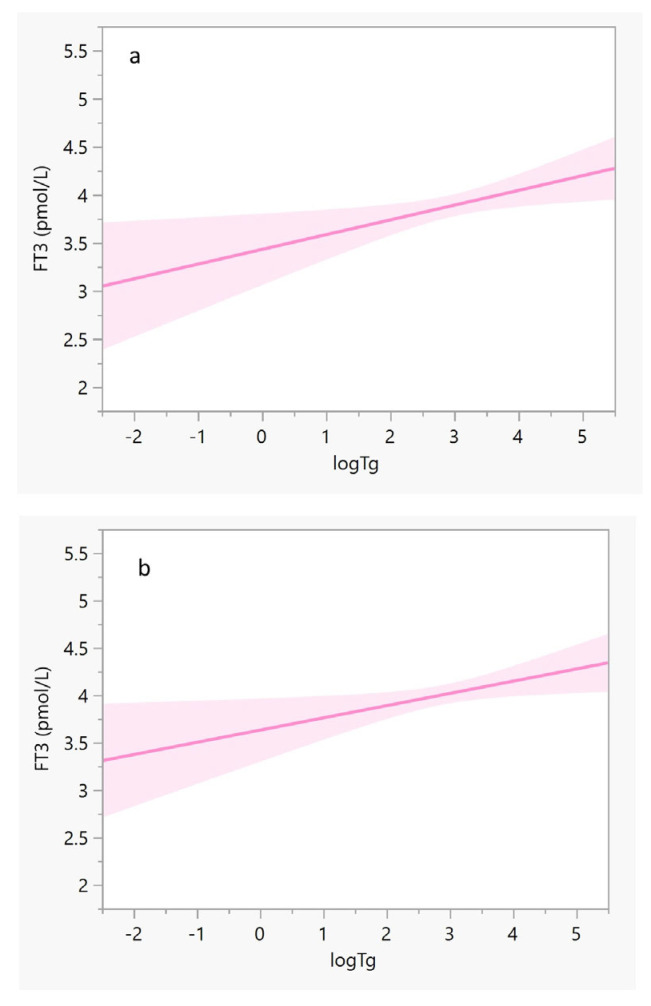
Regression lines of FT3 (*y*-axis) against log Tg (*x*-axis). The solid lines represent the estimated linear fit, and the shaded areas illustrate the 95% CIs. (**a**) Pregnant women in their third trimester (*N* = 138): y = 3.4 − 0.2(x), and R^2^ = 0.045; β (95% CI) = 0.19 (0.03, 0.27), *p* = 0.024; and (**b**) All pregnant women (*N* = 178): y = 3.6 − 0.1 × (x), and R^2^ = 0.032; β (95% CI) = 0.17 (0.02, 0.24), *p* = 0.024. After adjusting for participants’ age, smoking status, and gravidity, both correlations remained significant (*p* = 0.04 for both). Abbreviations: CI, confidence interval; FT3, triiodothyronine; log Tg, serum log-transformed thyroglobulin.

The iodine intake estimated by sIFFQ did not correlate with UIC levels. However, there was a statistically significant inverse association between iodine intake and Tg values among the study population (β = −0.2, F = 7.5, *p* < 0.01). Multivariate regression analysis showed that this association remained significant also after adjusting for age, smoking, and gravidity (β = (95% CI) = −0.198 (−0.0005, −0.003, *p* = 0.04, Figure 2). No correlation was observed between sIFFQ and UIC levels, thyroid function tests, or serum Tg, although iodine status measured by both UIC and serum Tg was significantly lower among participants with inadequate iodine intake (Table 2). Additionally, serum Tg values were significantly associated with FT3 values among all participants and those in the third trimester (Figure 3). The likelihood of having an intake below the EAR was almost three times higher in the upper FT3 decile, compared with the lower 1–9th deciles (OR = 2.6 [95% CI 1.1, 6.4], *p* = 0.04; Table 3). In contrast, women with FT3 levels in the upper decile were more than three times less likely to consume iodine-containing supplements (OR = 0.3 [95% CI 0.1, 0.8]; *p* = 0.01). These women also had higher Tg levels (*p* = 0.06), lower FT4 (*p* = 0.01), and a lower proportion of isolated hypothyroxinemia (*p* = 0.03) compared to those in lower FT3 deciles. Age, parity and BMI did not differ between groups.

### 3.3. Neonatal Iodine Status, Outcomes, and Thyroid Function

Of 178 newborns, 171 were born in BUMCA and had sufficient data for analysis. Among them, 57 newborns had appropriate data for nTSH analysis, as early postpartum discharge implementation was performed in BUMCA during the COVID-19 pandemic, and additional informed consent to collect samples from newborns was not obtained from all participants. The frequency of elevated nTSH in samples collected 3–4 days after birth was 16%. The distribution of gender and preterm delivery was similar among those who were born to mothers with FT3 levels in the uppermost decile compared to the others. Gestational age, Apgar scores, and anthropometrics percentiles were not statistically different among groups (Table 3). Mean neonatal TT4 and nTSH levels were similar between newborns to women, with FT3 values in the uppermost decile compared to the others. However, the proportion of newborns with nTSH above 20 IU/L was higher to a statistically significant degree among those who were born to women with FT3 values in the uppermost decile (Table 3).

## 4. Discussion

The current study is one of only a few describing an apparent association between increased FT3 values and inadequate iodine intake (Table 3) among a population of pregnant women with mild-to-moderate iodine deficiency (Table 1). This may support further analysis to examine whether insufficient maternal iodine status might alter FT3 rather than TSH and FT4 values, which are more common measurements for thyroid function. Our analysis shows that lower indices of estimated iodine intake and iodine-containing supplements are associated with presumably altered maternal thyroid function, including higher FT3 (Table 3). Furthermore, Tg values, an iodine status marker among euthyroid pregnant women, were positively correlated with FT3 values (Figure 3). However, we did not measure thyroid volume, assess placental function, or perform repeated thyroid function tests, which may have better isolated the impact of iodine deficiency on FT3 [2,9,22]. Thus, further insight into the link between thyroid function tests and iodine deficiency duration is warranted.

Due to the limitations mentioned above, the possible connection between iodine deficiency and FT3 should be interpreted cautiously and thoroughly. The study results demonstrate that intermediate-term (weeks to months) iodine intake in pregnancy, measured by Tg, may be associated with higher plasma FT3 values (Table 3) and might affect thyroid hormones differently than short-term and long-term iodine intake (estimated by the UIC and sFFQ, respectively). Unlike FT3 values, FT4 values were not found to be associated with any of the iodine status measures. The latter association contradicts the activity dynamics of type 2 deiodinase, which converts T4 to T3, and decreases as pregnancy progresses under normal circumstances [34]. Nevertheless, our findings are in line with several observations in Norwegian pregnant populations with insufficient iodine status [29,30]. This pattern of differentiated thyroid hormone alternation could be attributed to a dual mechanism. First, an autoregulatory loop preserves iodine in response to decreased availability of dietary iodine. A preferential conversion of T3 over T4 saves one iodine atom per molecule of hormone produced while simultaneously securing the availability of T3 to most maternal tissues [32]. Second, the significant elevation of FT3 (rather than FT4) during the third trimester in women with iodine deficiency may be related to possible subclinical or transient enlargement of the thyroid tissue due to long-term iodine deficiency, followed by elevated Tg. Indeed, most study participants were in their third trimester when Tg and thyroid hormones were measured. Moreover, the estimated iodine intake (measured by sIFFQ) was correlated with Tg values (Figure 2). It is noteworthy that the sIFFQ used in this study was intended to identify the average one-year iodine intake prior to recruitment [23].

Our study has found a significant correlation between sIFFQ levels and Tg values. Therefore, our study can strengthen the growing body of evidence by indicating the importance of Tg values in assessing maternal population iodine status [35]. Moreover, this finding may indicate that the iodine content of food items included in our sIFFQ possibly reflects the estimated iodine intake during the period of our study (2018–2020). However, our approximate estimate of iodine intake cannot be generalized to the entire population and may not reflect current food processing or agricultural practices in Israel or elsewhere [36]. It may be desirable to develop a rapidly adaptable and regional electronic tool for detecting iodine intake in public. Unlike Israel, other developed countries have professional software that may assist RDs in calculating iodine intake, although they cannot modify nutrition, and highlighting nutritional errors [9,10,11,12,16,29]. Accurately evaluating annual chemical iodine intake is close to impossible. Developing this type of software should be continuously updated and accompanied by frequent monitoring of changes in food iodine content (preferably more than once a year). We propose the use of such a method is necessary in light of ongoing population growth and climate change. Iodine content changes are apparent in agricultural and food preparation practices [18,19,36]. All in all, the approximate estimation of iodine intake in the present study substantially limits the generalizability of its findings.

The possible correlation between mild-to-moderate iodine deficiency and elevated maternal FT3 values deserves further investigation. This association is inconsistent with the results of a randomized controlled clinical trial in a region in Italy with mild-to-moderate iodine status, which did not find such an association [31]. The difference in FT3 values between the results of the inadequate iodine intake subgroup of the current study (Table 2) and the Italian study may be explained by the lower iodine status as reflected by both UIC and serum Tg medians (52.5 μg/L and 21.5 μg/L vs. 84.2 μg/g and 9.8 ng/mL, respectively) [31]. Another cross-sectional study that was carried out in central Israel (a region with mild-to-moderate iodine status) demonstrated mean serum Tg and FT3 of 16.6 ng/mL and 4.4 pmol/L, respectively, among a subgroup of 77 pregnant women in their first trimester. This subgroup’s iodine status (with UIC < 100 μg/L) had similar mean FT3 values to other subgroups with higher UIC [33]. The observed differences in FT3 values between the other Israeli study and the current study may stem from the different gestational stages examined (first vs. the third trimester), which reflect different iodine needs. It is noteworthy that in a Belgian cohort that examined the iodine status among 180 euthyroid pregnant women, the values of FT4 were normal, while serum Tg values were high, probably due to the goitrogenic effect of mild iodine deficiency [37]. Although thyroid gland volume was not measured in the current study, the significant correlation between serum Tg with both estimated iodine intake and FT3 values (Figure 2 and Figure 3) suggests investigating whether mild-to-moderate iodine deficiency during pregnancy may alter thyroid function.

The strengths of this study included a rigorous study design and data collection. The iodine intake assessment was performed by an experienced registered dietitian. All in all, iodine status in this mother–child matching pairs cohort was measured by a complementary model using four different approaches (sIFFQ, Tg, UIC, as well as nTSH). Additionally, outcomes were assessed by a trained and standardized team. Nevertheless, it is important to note that this study has several limitations. First, although the FFQ method captures iodine-rich sources that are not regularly consumed and, to some extent, accounts for day-to-day variation in overall consumption patterns, it is inaccurate in regards to quantifying the total amount of iodized salt consumed. A method such as 24-h food intake recall may seem to be an alternative; however, 24-h recalls generally underestimates salt consumption and normally measures only short-term intake [11]. Additionally, we used UIC to indicate recent days’ iodine intake. Second, the study sample is relatively small, which raises concerns about the possibility of type II errors (a low level of statistical power to detect the purported associations). However, the relatively high percentage of participants with FT3 values in the upper decile and UIC < 150 μg/L, miscarriage, and abnormal nTSH frequency, as well as other possible confounders, such as maternal and neonatal anthropometric indices, and gestational age, were similar across the FT3 deciles (Table 3). Third, it was a hospital-based study with a single geographical catchment area, so the results of this study cannot be generalized to all populations of pregnant women with mild-to-moderate iodine deficiency. While we were able to assess the contribution of maternal iodine status to maternal and neonatal thyroid function under certain assumptions, the relative lack of variance related to participants being from narrow iodine intake levels did not allow us to compare neonatal outcomes in populations with severe iodine deficiency or excess iodine intake. Fourth, the impact of type 2 deiodinase polymorphism, which may alter FT3 values, was not evaluated in this study. However, iodine deficiency might expose otherwise non-identified effects of genetic deiodinase variants. Moreover, studies that examined type 2 deiodinase polymorphism and increased FT3 values in different geographical areas (in which dietary factors such as iodine status may vary) did not reveal any apparent nor prevalent link [38]. Fifth, only 57 newborns had appropriate thyroid function test data for analysis. This low level of sampling is explained by the implementation of early postpartum discharge during the COVID-19 pandemic and ethical procedures requiring additional informed consent to collect samples from newborns. Lastly, the majority of participants were recruited in late pregnancy, and the difference in median gestational age was nearly significant between sub-groups (Table 3); therefore, we could not rule out longer exposure periods to better interpret birth outcomes, as well as gestational bias. However, to deal with recall bias regarding supplement daily intake, the sIFFQ used in this study was designed to estimate iodine intake in the year prior to recruitment and included questions on both initiation and time-frame consumption of iodine-containing supplements. Consequently, it should be noted that the prevalence of iodine-containing supplement intake and the estimated iodine intake from iodine-containing supplements in the current study were lower in sub-groups with presumably altered FT3 values (Table 3).

## 5. Conclusions

The present study highlights the probability that iodine status during pregnancy may be associated with maternal thyroid function. These initial findings indicate a possible association between iodine insufficiency and increased maternal FT3 in regions with mild-to-moderate iodine deficiency. If true, a higher contribution from iodine-containing supplements to the total iodine intake of pregnant women in these areas could be beneficial to their infants’ normal thyroid functioning. Since a modest sample size was used for this study, further analysis of reliable data is needed to uncover trends related to mild-to-moderate iodine deficiency during pregnancy, as well as both maternal and neonatal thyroid function.

## Figures and Tables

**Table 1:** Descriptive characteristics *^d^* of the study population.

Characteristics	Study Population *N* = 178
Age (years)	31 ± 5
Israeli born	147 (83%)
Tertiary education	136 (76%)
Alcohol consumption	1 (~0%)
Smoking	
Current smoker	25 (14%)
Past smoker	37 (21%)
Previous X-ray examinations	40 (22%)
Post-psychological stressful event	31 (17%)
Prior use of possible thyroid-disrupting medication	34 (19%)
Family history of TD	53 (28%)
BMI (kg/cm^2^)	
Preconception	24.1 ± 4.9
At recruitment	28.4 ± 5.2
Change	4.4 ± 2.6
Gestational week at recruitment	32.2 (27–35)
Parity	2.5 ± 1.6
Gravidity	3.2 ± 2.0
Estimated dietary iodine intake (μg/day)	179 (94–268)
Estimated iodine intake from ICS (μg/day) *^b^*	62 (0–150)
ICS initiation’s gestational age (week)	4 (0–8)
serum Tg (μg/L)	18 (11–33)
UIC (μg/L)	60 (41–95)
Pre-pregnancy ICS initiation *	9 (6%)
ICS intake *^a^*	108 (61%)
Iodized salt use ^#^	8 (5%)
Iodine intake < EAR	114 (64%)
Serum Tg > 13 (μg/L)	123 (69%)
Daily dietary goitrogens exposure	31 (17%)

*^d^* Categorical variables are shown as numbers and percentages in brackets; normally distributed continuous variables are presented as mean ± SD; continuous variables that did not distribute normally are shown as median and interquartile ranges in brackets. BMI, body mass index; ICS, iodine-contained supplement; EAR, estimated average requirements (160 μg/day); Tg, thyroglobulin; UIC, urinary iodine concentration, TD, thyroid disease; *^b^* based on participant reporting ICS intake; * Only participant who reported ICS initiation time (*N* = 157); *^a^* At recruitment; ^#^ Iodized salt, table salts with 3 μg of iodine per 100 g.

**Table 2:** Maternal iodine status and thyroid function tests *^a^* classified below and above EAR with the aid of sIFFQ for estimating iodine intake adequacy.

Characteristics	Adequate *N* = 85	Inadequate *N* = 114	*p*
Median UIC (IQR), μg/L *^b^*	70 (47–117)	51 (33–78)	0.03 *^c^*
Mean serum Tg ± SE, μg/L	15.8 ± 0.1	20.5 ± 0.1	0.07 *^d^*
Median serum Tg (IQR), μg/L	15.9 (10.4–31.1)	21.5 (12.2–38.3)	<0.01 ^*b*^
Median TSH (IQR), mIU/L	1.6 (1.1–2.1)	1.6 (1.1–2.1)	NS
Mean FT3 ± SD, pmol/L	4.0 ± 0.6	4.0 ± 0.8	NS
Mean FT4 ± SD, μg/L	1.0 ± 0.1	1.0 ± 0.2	NS
Mean FT3/FT4 ratio ± SD	3.9 ± 0.7	4.0 ± 0.8	NS

*^a^* Continuous results that were not distributed normally were summarized by median and IQR. Normally distributed continuous parameters were presented as mean ± SD; *^b^ N* = 165; *^c^ p*-value by median test; *^d^* Geometric mean ± SE; EAR, estimated average requirements (160 μg/day); sIFFQ, semi-quantitative iodine food frequency questionnaire; FT3, Free triiodothyronine; FT4, thyroxine; IQR, interquartile range; NS, not significant; SE, standard error; SD, standard deviation; Tg, thyroglobulin; TSH, thyrotropin; UIC, urinary iodine concentration.

**Table 3:** Iodine-related dietary characteristics and thyroid function tests *^d^* of pregnant women and their newborn pregnancy outcomes and newborn anthropometrics by FT3 deciles.

	FT3 Deciles		
	1–9 *N* = 154	10 *N* = 24	*p*-Value
**Pregnant Women**			
Gestational week at recruitment	34 (27–37)	29 (26–32)	0.06
Iodine Intake			
Estimated median dietary Iodine intake, μg/day (IQR)	189 (102–272)	101 (60–280)	NS
Iodine intake < RDA, *N* (%)	95 (58%)	18 (75%)	NS
Iodine intake < EAR, *N* (%) *^F^*	60 (39%)	15 (63%)	0.0
Iodized salt use, *N* (%) ^#^	8 (8%)	0 (0%)	NS
ICS intake, *N* (%) *^F^*	99 (66%)	9 (38%)	0.01
Estimated iodine intake from ICS (μg/day), median (IQR)	95 (0–154)	0 (0–150)	0.04
Dietary goitrogens exposure, *N* (%) *^F^*	31 (19%)	0 (0%)	0.02
Serum Tg			
Median Serum Tg, μg/L (IQR)	17 (11–32)	24 (14–48)	0.08
Participants with Tg > 40 μg/L, *N* (%)	26 (16%)	6 (25%)	NS
UIC			
Median UIC, μg/L (IQR) ^A^	61 (42–98)	42 (21–75)	NS
Participants with UIC < 150 μg/L, *N* (%) *^L^*	130 (87%)	16 (100%)	0.04
TSH			
Mean TSH ± SD, mIU/L	1.7 ± 0.9	2.1 ± 1.0	NS
Participants with subclinical hypothyroidism, *N* (%)	4 (2%)	2 (8%)	NS
FT4			
Mean FT4 ± SD, μg/L *	1.0 ± 0.1	0.9 ± 0.2	0.01
Participants with isolated hypothyroxinemia, *N*%) *^L^*	16 (10%)	0 (0%)	0.03
TPO Ab, *N* (%) *^Pe^*	7 (4%)	1 (4%)	NS
Tg Ab, *N* (%)	3 (1%)	2 (8%)	NS
**Newborns at birth, *N* (%)**	149	22	
At birth			
Mean gestational age ± SD, days	269 ± 17	249 ± 59	NS
Preterm birth, *N* (%)	21 (14%)	6 (27%)	NS
Gender (Female, Male)	70, 79	4, 15	NS
Mean Apgar score ± SD			
At 1 min after delivery	8.9 ± 0.8	9.0 ± 0.3	NS
At 5 min after delivery	9.9 ± 0.4	10 ± 0.0	NS
Mean weight percentile ± SD *^Do^*	52.5 ± 27.7	51.6 ± 24.1	NS
Mean length percentile ± SD *^Da^*	71.9 ± 26.1	78.5 ± 24.7	NS
Mean head circumference ± SD	34.2 ± 1.9	34.2 ± 1.7	NS
Mean total T4 ± SD, nmol/L	15.2 ± 3.8	15.8 ± 4.9	NS
Median nTSH (IQR), IU/L	5.0 (3.3–7.9)	2.0 (0.5–6.9)	NS
Newborns with elevated nTSH ≥ 5 IU/L, *N* (%)	29 (53%)	1 (50%)	NS
Newborns with elevated nTSH ≥ 20 IU/L, *N* (%) ^*T*^	0 (0%)	1 (50%)	0.04

*^d^* Continuous results that were not distributed normally were summarized by the median and interquartile range (IQR). Normally distributed continuous parameters were presented as mean ± standard deviation (SD); FT3, Free triiodothyronine; NS, not significant; RDA, recommended daily allowance (220 μg/day); EAR, estimated average requirement (160 μg/day); ICS, iodine-containing supplement; IQR, interquartile range; Tg, thyroglobulin; TSH, thyrotropin—thyroid stimulating hormone; nTSH, neonatal TSH; subclinical Hypothyroidism (TSH > 2.5 mU/L during 1st or 4.0 mU/L in both 2nd and 3rd trimesters) [2]; UIC, urinary iodine concentration; SD, standard deviation; FT4, free thyroxine; Isolated hypothyroxinemia (FT4 < 0.93 ng/L, TSH > 2.5 mU/L or 4.0 mU/L during 1st and both 2nd 3rd trimester; TPO Ab, thyroid peroxidase antibodies; Tg Ab, thyroglobulin antibodies. Iodized salt, 3 μg iodine/100 gr; *^Do^* Adjusted for gestational age and gender according to birth weight Israeli standards [28]; *^Da^* Adjusted for birth week and gender according to birth length and head circumference Israeli standards [27]. *^F^* Significant difference (Fisher’s Exact test, α = 0.05); ^#^ Iodized salt, table salts with 3 μg of iodine per 100 g; ^A^ For only participants with available UIC measurements (*N* = 165); *^L^* Significant difference (Likelihood ratio test, α = 0.05); * Significant difference (Student’s *t*-test, α = 0.05); *^Pe^* Significant difference (Pearson test, α = 0.05); *^T^* For only term newborns sampled for thyroid function > 48 h after birth (*N* = 57).

## Data Availability

Data described in the manuscript, code book, and analytic code will be made available upon request pending application and approval.
